# The role of lymphatic endothelial cell metabolism in lymphangiogenesis and disease

**DOI:** 10.3389/fcvm.2024.1392816

**Published:** 2024-05-09

**Authors:** Summer Simeroth, Pengchun Yu

**Affiliations:** ^1^ Cardiovascular Biology Research Program, Oklahoma Medical Research Foundation, Oklahoma City, OK, United States; ^2^ Department of Cell Biology, University of Oklahoma Health Sciences Center, Oklahoma City, OK, United States

**Keywords:** lymphatic vessel, lymphatic endothelial cell, cellular metabolism, metabolic enzyme, lymphatic disease, lymphatic development, lymphangiogenesis

## Abstract

Lymphatic endothelial cells (LECs) line lymphatic vessels, which play an important role in the transport of lymph fluid throughout the human body. An organized lymphatic network develops via a process termed “lymphangiogenesis.” During development, LECs respond to growth factor signaling to initiate the formation of a primary lymphatic vascular network. These LECs display a unique metabolic profile, preferring to undergo glycolysis even in the presence of oxygen. In addition to their reliance on glycolysis, LECs utilize other metabolic pathways such as fatty acid β-oxidation, ketone body oxidation, mitochondrial respiration, and lipid droplet autophagy to support lymphangiogenesis. This review summarizes the current understanding of metabolic regulation of lymphangiogenesis. Moreover, it highlights how LEC metabolism is implicated in various pathological conditions.

## 1 Introduction

Lymphatic vessels (LVs) absorb interstitial fluid from the surrounding tissues and transport it as lymph fluid, which contains immune cells, proteins, and dietary fats, back to the venous circulation. If LVs are impaired or dysfunctional, excessive interstitial fluid cannot be properly drained, causing accumulation in tissues leading to painful swelling, a disease termed lymphedema. Additionally, LVs are also involved in inflammation, graft rejection, myocardial infarction, and many other disease conditions (1–6). Therefore, elucidating how LVs are formed will further the understanding of physiological and pathological processes regulated by lymphatics. Moreover, it will facilitate the development of new treatments for lymphatics-associated diseases.

**Figure 1:**
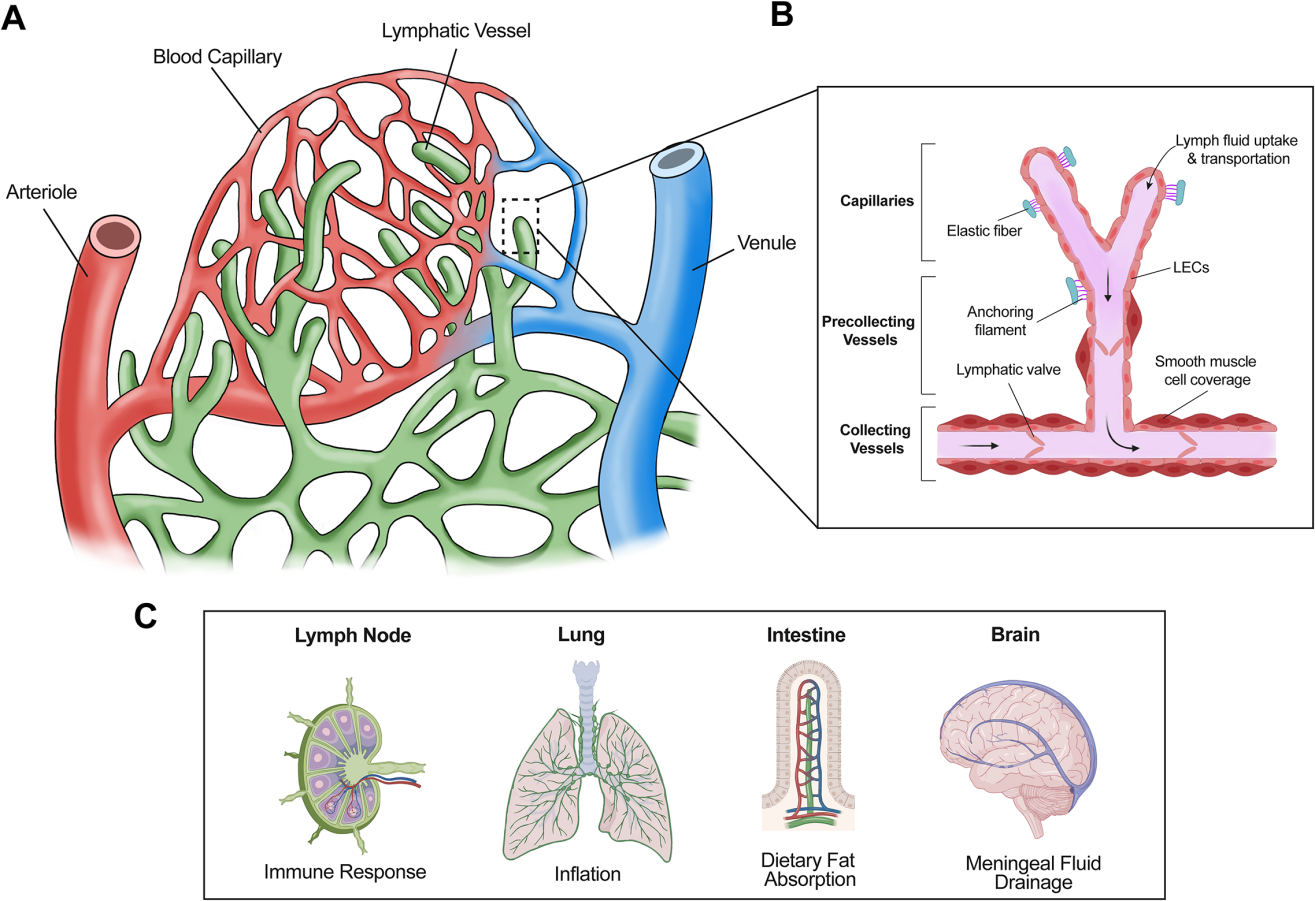
Structure and general functions of lymphatic vessels. (**A**) The lymphatic system absorbs interstitial fluid from the surrounding tissue bed for transport as lymph. (**B**) Initial fluid uptake is achieved through lymphatic capillaries through button like junctions that separate to allow fluid entry. The lymph is then passed to the precollecting and collecting lymphatic vessels, which have smooth muscle cell coverage and lymphatic valves, thus facilitating unidirectional transport. (**C**) Lymphatic vessels are present in multiple tissues and play important roles in several physiological processes. For example, lymphatic vessels facilitate immune response via immune cell trafficking in the lymph nodes, aid in inflation of the lung, absorb dietary fats via lacteals in the intestine, and assist with cerebrospinal fluid drainage in the meninges.

The primary constituents of the lymphatic vasculature are lymphatic endothelial cells (LECs), which line lymphatic capillaries and collecting LVs (7). Absorption of initial fluid from surrounding tissue is achieved by lymphatic capillaries, which are blunt-ended vessels composed of a single layer of LECs (8). These LECs are interconnected by button-like junctions with a discontinuous basement membrane, thus allowing anchoring filaments to facilitate fluid uptake into lymphatic capillaries via interaction with the extracellular matrix (8, 9). Upon entering the lymphatic vasculature, the fluid absorbed from surrounding tissues is termed “lymph” (10). Lymph carries immune cells and pathogens and is involved in adaptive immune response in the lymph node (11). Moreover, dietary lipids, cholesterol, and macromolecules are taken up by lymphatic capillaries and transported as part of lymph in LVs (12). Therefore, in addition to immune response, LVs are involved in several important physiological and pathological processes (12) (Figure 1). For example, in the intestine, lymphatic capillaries (lacteals) absorb dietary fats incorporated into chylomicrons for transport to the bloodstream (13). Thus, lymphatic dysfunction is associated with obesity and insulin resistance (14). LVs also play a role in reverse cholesterol transport, a process that can ameliorate atherosclerosis (15). In the lung, lymphatic drainage is required during neonatal development of the lung allowing for proper inflation (16). Additionally, meningeal LVs assist in clearing macromolecules in cerebrospinal fluid (17, 18).

After the interstitial fluid enters lymphatic capillaries via button junctions, it flows towards collecting LVs (Figure 1), which possess zipper junctions in contrast to button junctions (8). These unique zipper junctions form a tighter, more continuous structure with a complete basement membrane to limit the improper entry or exit of lymph fluid (8). Collecting LVs serve a special purpose within the context of fluid transport. Specialized smooth muscle cell coverage and lymphatic valves allow collecting LVs to act as the motor unit of the lymphatic system, propelling lymph fluid unidirectionally towards the lymph nodes where it will eventually converge in the thoracic duct before returning to the circulatory system via the subclavian vein (19).

During early development, blood vascular endothelial cells differentiate *in situ* from a population of endothelial cell progenitors known as angioblasts and form the primitive vascular plexus, a process termed vasculogenesis (20). This primary vessel network further expands through angiogenesis, during which new blood vessels sprout from existing vessels (20). Under the direction of signaling pathways such as Notch and vascular endothelial growth factor (VEGF) signaling, blood vascular endothelial cells differentiate into arterial and venous endothelial cells, which respectively form arteries and veins that function together to support oxygen exchange and transportation of nutrients and waste (21). In parallel with blood vascular development, lymphatic vascular formation begins with LEC differentiation (22). The first lymphatic structures, the primary lymph sacs, develop from the embryonic cardinal veins (23). After the development of the primary lymph sacs, new LVs continue sprouting, branching, and maturing to form a more extensive LV network (24). LEC differentiation and subsequent LV expansion are controlled by two critical drivers—PROX1 and VEGF receptor 3 (VEGFR3) (23, 25). The transcription factor PROX1 is a master driver of LEC differentiation, and its expression is regulated by COUP-TFII and SOX18 signaling (26, 27). PROX1 promotes the expression of VEGFR3 in LECs, which mediates the effect of VEGF-C in stimulating LEC sprouting, migration, and proliferation during LV development (28, 29).

In addition to PROX1–VEGFR3 signaling, several other molecules, particularly cellular metabolism regulators, have recently been identified as crucial drivers of LV formation. In this review, we will summarize our understanding of LV formation from the perspective of cellular metabolism and explore how this understanding may offer new therapeutic strategies for human pathological conditions.

## 2 Cellular metabolic pathways in lymphangiogenesis

### 2.1 Glycolysis

LECs generate nearly 70% of ATP via glycolysis instead of other metabolic processes such as oxidative phosphorylation, a unique characteristic differentiating LECs from many other cell types (30, 31). In addition to energy generation, glycolysis can also be exploited as a means of biomass production, which supports cell proliferation and growth (32). The distinctive reliance of LECs on glycolysis even in the presence of oxygen is mirrored by the Warburg Effect in cancer cells (32).

The rate-limiting steps in glycolysis are under the control of specific enzymes that aid in the regulation of metabolism, such as hexokinases (HKs) which convert glucose to glucose-6-phosphate in the first step of glycolysis (33, 34). In mammals, there are four HK isozymes: HK1, HK2, HK3, and HK4 (30). These HK isozymes differ in their regulatory properties, localizations, and enzymatic activities. More specifically, the activity of HK1, HK2, and HK3 is generally feedback inhibited by the presence of high glucose-6-phosphate levels whereas HK4 activity does not commonly exhibit this limitation under similar conditions (33). Furthermore, HK1 and HK2 are physically linked to the mitochondria in contrast to HK3 and HK4 (33). The association with the mitochondria is necessary for HK2-mediated proliferation of hepatocellular carcinoma cells, as disruption of this binding inhibits tumor growth and induces apoptosis (35). Moreover, the binding of HK2 to the mitochondria aids in the protection against oxidative stress and cell death (36, 37). Despite these studies, it remains unclear whether the physical association between HK2 and the mitochondria is important for LEC proliferation and survival.

Functional roles of HK2 in LV development have been recently implicated using genetic mouse models. Genetic deletion of *Hk2* in mice results in impaired formation of the primitive lymphatic vascular plexus during early embryonic development (38). More specifically, embryos that underwent LEC-specific *Hk2* knockout displayed attenuated LV sprouting and migration towards the midline in the dorsal skin as early as embryonic day (E) 15.5 (38). Consistently, *Hk2* knockout in LECs suppresses fibroblast growth factor 2 (FGF2)-induced lymphangiogenesis in the adult mouse cornea (38). Moreover, pan-endothelial deletion of *Hk2* produced embryos with impeded arterial development and angiogenesis in the embryonic skin, supporting that HK2 contributes to both angiogenesis and lymphangiogenesis (38). Another regulator of endothelial cell metabolism and angiogenesis is 6-Phosphofructo-2-kinase/fructose-2, 6-bisphosphatase, isoform 3 (PFKFB3) which acts to synthesize fructose-2,6-bisphospate (F2,6P_2_), an important allosteric activator of 6-phosphofructo-1-kinase (PFK-1) (31). During glycolysis, PFK-1 controls the rate-limiting conversion step of fructose-6-phosphate (F6P) to fructose-1,6-bisphosphate (F1,6P_2_) (31). When PFKFB3 is ablated in murine models, developmental angiogenesis in the retina and the hindbrain as well as pathological retinal neovascularization are all impaired, demonstrating the importance of glycolysis in endothelial cell growth and vessel formation (31, 39). In addition to PFKFB3 and HK2, recent findings have also demonstrated the importance of pyruvate kinase type M2 (PKM2) for lymphangiogenesis (40). PKM2 converts phosphoenol pyruvate to pyruvate, generating ATP at the end of the glycolytic pathway (41). Thus, as a main driver of glycolytic ATP production, PKM2 has been linked to lymphangiogenic processes of LECs, and treatment with shikonin, which non-specifically inhibits PKM2 and several other molecules, alleviates lymphatic lesions induced by lipopolysaccharides in rats (40).

Furthermore, glycolysis has been shown to play a functional role in pathologies relating to abnormal pericyte function (42). Pericytes, also defined as mural cells, are a specialized cell type that envelops the endothelial cells that make up the capillary system (43, 44). Pericytes embed themselves within the basement membrane of endothelial cells, facilitating a close interaction between the microvasculature and mural cells (43). Pericytes are recruited to the microvasculature during development via cell signaling pathways, notably utilizing factors such as platelet-derived growth factor B (PDGF-B) and its receptor, PDGF receptor β (PDGFRβ) (45–48). Specifically, PDGF-B is secreted by endothelial tip cells along the angiogenic front during vessel formation, which then attracts pericytes expressing PDGFRβ, aiding in vasculature maturation (43). Functionally, the recruitment of pericytes is important in mediating blood flow as they contribute to regulating both vasoconstriction and vasodilation (49). Pericytes have recently become a topic of interest in the study of pathological tumor growth as vasculature within tumors has demonstrated dysregulation in pericyte-endothelial cell interactions leading to abnormal blood vessel sprouting (42, 50). Additionally, abnormal pericyte death contributes to diabetic retinopathy, as altered blood flow in the retina contributes to vascular leakage resulting in decreased vision acuity (51–56). Interestingly, HK2 has proven to be critical for pericyte contractility during tumor angiogenesis (42). HK2 is a main driver of tumor pericyte glycolysis, which (via the ROCK2-MLC2 pathway) increases pericyte contractility, resulting in impaired blood flow in tumors (42). Thus, inhibition of HK2 activity using a non-specific inhibitor remodels tumor vasculature and enhances the delivery and efficacy of a chemotherapy drug (42). However, it is still unknown what instigates aberrant pericyte glycolysis in tumors and whether genetic ablation of HK2 in pericytes can impact tumor vasculature.

HK2 is regulated by MYC expression, which in turn is regulated by FGF signaling in LECs (38). Therefore, FGF signaling activation elevates, while its inhibition suppresses, glycolysis in LECs (38). Moreover, genetic ablation of FGF receptor 1/3 or MYC attenuates retinal angiogenesis and the formation of the primitive LV network in the embryonic skin, similar to the effect of *Hk2* knockout in LECs (38, 57). Interestingly, although MYC regulates the expression of multiple glycolytic enzymes in cancer cells (58), FGF-MYC signaling preferentially controls the expression of HK2, not HK1 or other rate-limiting glycolytic enzymes in LECs (38). Based on these results, it can be concluded that the decrease in FGF signaling results in a reduction of MYC, which markedly reduces HK2-driven glycolysis, causing subsequent defects in lymphangiogenesis (38). Despite this progress, many important questions remain to be addressed. For example, how FGF signaling activation upregulates MYC expression in LECs is unknown. Moreover, it is also unclear how FGF-MYC signaling selectively controls HK2 over the other rate-limiting glycolytic enzymes in LECs.

### 2.2 Fatty acid β-oxidation (FAO)

FAO is a biochemical process in which fatty acids are converted to acetyl-CoA, which is then oxidized via the tricarboxylic acid (TCA) cycle and the electron transport chain (ETC) (59). First, fatty acids enter the cell via cell surface transport molecules (59). Fatty acid translocase (FAT/CD36), membrane-bound fatty acid binding proteins (FABP), and fatty acid transport proteins (FATP) that are specific to the tissue are cell surface proteins that allow fatty acids to enter (59). Once inside the cell, fatty acyl-CoA synthase (FACS) adds a CoA group to the fatty acid, thus forming long-chain acyl-CoA (60). Carnitine palmitoyltransferase (CPT)1, which resides on the outer mitochondrial membrane, then converts long-chain acyl-CoA to long-chain acylcarnitine (60). This step is followed by transportation of long-chain acylcarnitine into the mitochondrial matrix, which is mediated by the action of carnitine-acylcarnitine translocase (60). CPT2, located on the inner mitochondrial membrane, then converts long-chain acylcarnitine back to long-chain acyl-CoA, which goes through β-oxidation to generate acetyl-CoA (60).

The importance of FAO for LV development has been revealed using mice deficient in CPT1A, which is the most abundant CPT1 isoform in LECs, and the CPT1 inhibitor etomoxir (61). *Cpt1a* deletion in LECs results in impaired and disorganized lymphatic growth towards the dorsal midline and a reduction in filopodia number, coupled with edema and blood-filled lymphatics in mouse embryos (61). Pharmacological inhibition of CPT1 via etomoxir inhibited FAO during early-stage development, which decreased the number of PROX1-expressing cells in the cardinal vein during early LEC differentiation, leading to defective dermal LV formation, reduced filopodia formation at the lymphangiogenic front, and a reduction in LEC proliferation (61). However, it is important to note that the activity of etomoxir may be independent of CPT1 activity under certain biological contexts, as displayed in cancer cell proliferation and T cell formation (62, 63). Additionally, fatty acid transporter CD36 has been shown to be expressed in the lacteals of intestinal lymphatics, which act to transport dietary lipids through the lymphatic system for subsequent entry to the subclavian vein (64, 65). Deletion of *Cd36* in a murine model caused discontinuous VE-cadherin junctions in lacteals (65), a process that has been shown to play a key role in chylomicron transport and diet-induced obesity (66). Thus, *Cd36* deficiency leads to LV leakage, late-onset obesity, and an increased risk of developing type 2 diabetes (65). Metabolically, silencing CD36 in LECs causes a reduction in FAO corresponding to an increase in glycolysis (65).

FAO exerts epigenetic control through histone acetylation (67). Histone acetylation occurs via the acetylation of lysine residues present on histones, during which acetyl groups act on the charged lysine residues to reduce the interaction between the histones and DNA, thus resulting in an open conformation that allows for the recruitment of various effectors that influence gene expression (68). Acetyl-CoA produced during FAO can be utilized as a substrate for histone acetylation (69). Histone acetyltransferases rely on acetyl-CoA levels produced via FAO and glycolysis to exert transcriptional control, and in glucose-limited environments, FAO is the main contributor of acetyl-CoA (67). First reported in 2009, ATP citrate lyase (ACL) converts glucose-derived citrate to acetyl-CoA when the appropriate nutrients are available, and deletion of ACL has been shown to decrease the expression of several glycolytic genes, providing further evidence that acetyl-CoA aids in transcriptional control (70). In this manner, acetyl-CoA has been shown to be an important regulator of target gene epigenetic modification by manner of histone acetylation (67). P300, a member of the histone acetyltransferase family, aids in epigenetic control via acetylation of histones, modifying core histones within the nucleosome allowing for transcriptional activation (71). FAO-derived acetyl-CoA fuels the activity of P300, which is recruited to the VEGFR3 promoter region together with PROX1 (61, 72). Thus, acetyl-CoA is utilized by P300 to exert transcriptional control by interacting with the PROX1-P300 complex, promoting acetylation of PROX1 target genes to control their transcription (61). Additionally, acetyl-CoA/CoA ratios determined by histone acetylation via P300 increased when PROX1 was overexpressed (61). Therefore, acetyl-CoA derived from FAO promotes PROX1-P300 expression which drives VEGFR3 transcription during lymphatic vascular development (61). In addition to supporting epigenetic regulation, acetyl-CoA generated from FAO contributes to the synthesis of deoxyribonucleotide triphosphate (dNTP) in endothelial cells (73). Isotopic labeling demonstrates that fatty acid carbons fuel the TCA cycle, contributing to the building of biomass, including dNTP (73). Therefore, genetic ablation of CPT1A suppresses endothelial proliferation *in vitro* and during vascular development in mice (73).

As previously mentioned, LECs utilize glycolysis to generate most of their ATP, and it is known that this process is further promoted via FGFR signaling during lymphangiogenesis (38). However, cells can shift their preferred metabolic pathway in response to stimuli that cause changes in energy demands—a term coined “metabolic flexibility” (74). Recently it has been found that inhibition of FGFR signaling-driven glycolysis in LECs upregulates CPT1A expression and FAO (74). As such, FAO, which makes little contribution to LEC ATP production under normal conditions, plays an important role in energy production and compensates for the deficit in energy when glycolysis is deficient (74). This process is mediated by peroxisome proliferator-activated receptor alpha (PPARα), which is upregulated upon FGFR inhibition and in turn, activates *CPT1A* transcription (74).

### 2.3 Ketone body oxidation (KBO)

KBO is critical in maintaining metabolic homeostasis in organisms, supporting the TCA cycle and thus mitochondrial respiration, especially under nutrient-poor conditions (75). KBO has been identified as a potential therapeutic target for several neurodegenerative diseases such as Alzheimer's and Parkinson's (76). 3-oxoacid CoA-transferase 1 (OXCT1) acts as one of the key regulators of KBO by catalyzing a rate-limiting step. KBO eventually leads to the production of two molecules of acetyl-CoA, which can subsequently enter into the TCA cycle (77). Other key enzymes include BDH1 (3-hydroxybutyrate dehydrogenase 1), mThiolase, and citrate synthase (78). In KBO, ketone body β-hydroxybutyrate (β-OHB) can be converted to acetoacetate via BDH1 (78). Acetoacetate can then be catalyzed to form acetoacetyl-CoA via OXCT1, which will go on to produce acetyl-CoA following mThiolase catalytic cleavage (77, 78). This cleavage event produces two acetyl-CoA molecules for entry into the TCA cycle controlled by citrate synthase (77, 78). Among ketone bodies, β-OHB is the most commonly found in the circulating bloodstream, which LECs can readily access (78). Inhibition of KBO via depletion of OXCT1 or BDH1 suppresses proliferation, migration, and spouting of LECs *in vitro* (78). Moreover, genetic deletion of *Oxct1* in LECs impedes LV development in the dorsal skin of mouse embryos (78). These data suggest that KBO is an important mechanism regulating LV growth (78). Furthermore, OXCT1 knockdown lowers the acetyl-CoA/CoA ratio and the amount of TCA cycle intermediates produced (78), therefore revealing the metabolic mechanisms by which KBO impacts LV development.

### 2.4 Mitochondrial respiration

The mitochondrial respiratory chain is made up of various complexes that shuttle electrons across the mitochondrial membrane to produce a proton energy gradient as hydrogen atoms are pumped into the intermembrane space, eventually fueling the phosphorylation of ATP from ADP by complex V, also known as ATPase (79). Among these complexes is complex III, which shuttles electrons to the inner mitochondrial-associated protein cytochrome c, which then delivers the electrons to complex IV (79). Complex III is made up of various subunits that assist in this trafficking process (80). One such subunit is ubiquinol-cytochrome c reductase complex III subunit IV (QPC) encoded by the *Uqcrq* gene (80). Mutant QPC mice display a downregulation of lymphatic markers leading to an attenuation in LV development (81). Chemical inhibition of complex III also causes reduced methylation at the genetic loci of *Vegfr3* and *Prox1* (81). The resulting downregulation of PROX1 in LECs causes a reduction in LEC fate specification, which is further exacerbated by the reduction of VEGFR3 that is mediated via PROX1 in the feedback-loop system necessary to retain LEC fate identity (81). Mitochondrial respiration also plays a role in regulating the NAD^+^ (oxidized nicotinamide adenine dinucleotide)/NADH (reduced nicotinamide adenine dinucleotide) ratio, nucleotide synthesis, and levels of several TCA cycle intermediates, such as citrate, fumarate, and malate (81). Moreover, inhibition of complex III changes H3K4 methylation and H3K27 acetylation at the *VEGFR3* locus (81), offering evidence that epigenetic modifications of *Vegfr3* are critically mediated by complex III of the mitochondria (81).

### 2.5 Autophagy of lipid droplets (LDs)

Autophagy, a conserved lysosomal degradation and cellular recycling pathway, has been implicated as playing an important role in the blood vasculature and the maintenance of cellular metabolic homeostasis (82–84). Specifically, autophagy allows the cell to recycle metabolites to use as fuel for metabolism or to support biosynthesis (82, 83). Similarly, autophagy has recently been identified as an active mechanism in supporting lymphangiogenesis (85). This is accomplished via the autophagy of LDs, a process known as lipophagy (85). LDs are unique organelles found in most cell types that store neutral lipids surrounded by a phospholipid monolayer which can interact and associate with various organelles including the mitochondria to influence cellular metabolism (86). Furthermore, LDs sequester fatty acids in the form of triacylglycerol to be used as metabolic fuel during cell growth or when nutrient levels are depleted (87). In LECs, lipophagy supports the trafficking and release of lipids to the mitochondria to maintain FAO (85). Not only does lipophagy influence LEC metabolism, but it also promotes the expression of lymphangiogenic markers such as PROX1 and VEGFR3 (85). Furthermore, lipophagy has also been identified as playing a role in pathological lymphangiogenesis, as genetic depletion of essential autophagy gene *Atg5* in mice LECs caused reduced lymphangiogenesis in a corneal wound healing model (85). Thus, autophagy of LDs in LECs represents a critical molecular mechanism supporting metabolic homeostasis, lymphangiogenesis, and its related gene expression (85).

## 3 Role of LEC metabolism in pathological lymphangiogenesis and its implication for human diseases

### 3.1 Lymphedema

Lymphedema is characterized by the accumulation of interstitial fluid resulting in impaired movement and often painful swelling in patients and can occur in various locations in the human body, most notably the limbs (88). It is often characterized as a chronic disease and is classified as either primary lymphedema or secondary lymphedema (88). Primary lymphedema is an inherited condition often caused by a genetic mutation that causes lymphatic formation defects, affecting 1 in every 100,000 people (88). More commonly, lymphedema arises as a result of damage, infection, or injury to the lymphatic system (88, 89). This is known as secondary lymphedema and affects 1 in 1,000 people in the United States alone (88). Notably, individuals who have undergone treatment for breast cancer or other gynecological cancers have an increased risk of developing secondary lymphedema, as 1 in 5 breast cancer survivors have been reported to develop this disease due to lymph node dissection and other cancer therapeutic techniques (90, 91). Although lymphedema is prevalent in the population, progressive, and often persists throughout the patient's life, therapeutic measures primarily consist of lymph massage, compression, exercise, and various microsurgical techniques with limited success (88, 92, 93). Thus, the need for drug therapies that target lymphedema is critical.

A tail injury mouse model of lymphedema that recapitulates human secondary lymphedema can be used as a method to explore what may influence the pathology of the disease (94). This is achieved by surgically removing LVs within the tail and then examining edema post-injury (94–96). As aforementioned, KBO has been found to promote lymphangiogenesis as LVs can readily access circulating β-OHB (78). Accordingly, it was found that mice that were fed the high-fat low-carb ketogenic diet (HFLC-KD) displayed less swelling in a tail injury model, and that ketone bodies aid in anti-inflammatory mechanisms by affecting leukocytes directly via β-OHB supplementation (78). Furthermore, mice fed the HFLC-KD also displayed reduced dermal thickening, decreased lymphatic dilation, and improved transport of lymph to the lymph node (78). Thus, targeting KBO shows promise in ameliorating lymphedema and promoting lymphangiogenesis as a potential future therapeutic measure.

### 3.2 Corneal graft rejection

The cornea, the outer clear layer of the eye that serves to refract light, is largely avascular (97). The absence of blood and lymphatic vasculature from the cornea maintains transparency, which is crucial for proper vision functionality (98, 99). The maintenance of this avascularity is established early in life, and under normal conditions is maintained throughout adulthood (100). However, in pathological conditions inflammation can cause the expansion of LVs and blood vessels into the avascular cornea (101). This aberrant lymphangiogenesis and angiogenesis can cause a decrease in the transparency of the cornea and loss of visual acuity (101). Furthermore, lymphangiogenesis into the cornea increases the risk of corneal graft rejection, a process that is mediated by VEGF-C/VEGFR3 signaling (4, 102–107). As many as ∼20% of patients who receive a corneal graft will face symptoms associated with rejection, with endothelial rejection being the most common cause (108).

Because of the close relationship between lymphangiogenesis and corneal graft rejection, corneal models of injury-induced lymphangiogenesis have become a valuable way of investigating the role of lymphatic metabolism in pathological conditions (109). As such, FAO has emerged as one contributing metabolic pathway (61). It has been found that in a corneal injury mouse model the inhibition of the key FAO enzyme CPT1 via etomoxir reduced lymphangiogenesis (61). Likewise, glycolysis has also been implicated in the control of corneal lymphangiogenesis (38). When pellets that released FGF2 were implanted into the cornea, mice that underwent LEC-specific deletion of the key glycolytic enzyme HK2 displayed decreased lymphangiogenesis in the cornea (38). Additionally, galectin-8, a carbohydrate-binding protein that promotes adhesion and cell motility of LECs, has been found to promote pathological lymphangiogenesis in the cornea (110). Specifically, galectin-8 is upregulated in both mouse and human corneas following corneal inflammation, and increased galectin-8 escalates the rate of graft rejection in a mouse model of corneal transplantation (110). Consequently, inhibition or knockout of galectin-8 has shown promise in ameliorating lymphangiogenesis in the cornea *in vivo* (110). These results point towards potential therapeutic targets that may be investigated further for their role in inflammation-induced lymphangiogenesis contributing to graft rejection.

## 4 Discussion

**Figure 2:**
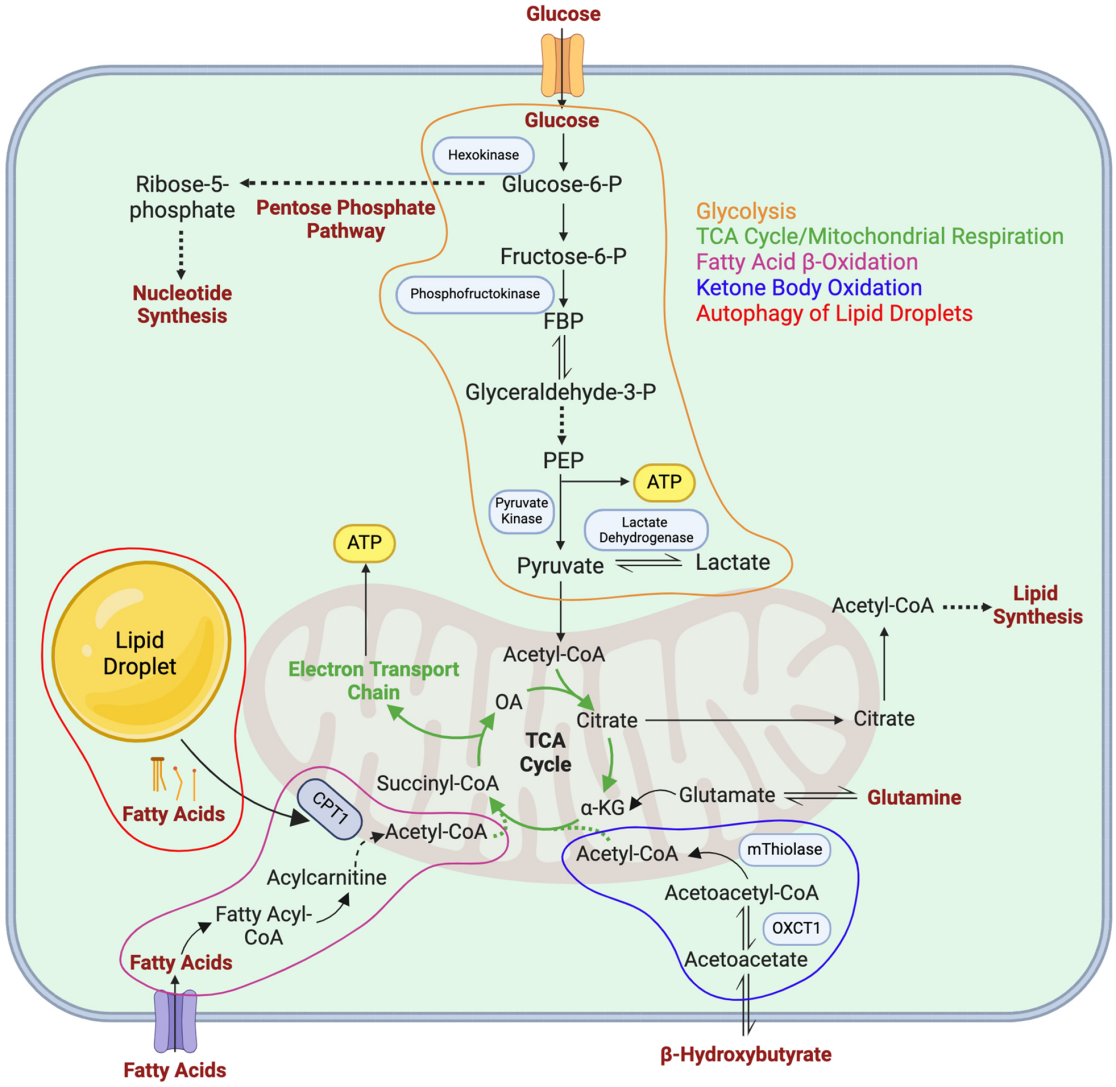
Metabolic pathways in LECs. Major metabolic pathways utilized by LECs as described in detail in the main text are depicted in orange (glycolysis), green (mitochondrial respiration/the TCA cycle), purple (fatty acid β-oxidation), blue (ketone body oxidation), and red (autophagy of lipid droplets). FBP, fructose 1,6-bisphosphate; PEP, phosphoenolpyruvate carboxylase; α-KG, α-ketoglutarate; OXCT1, 3-oxoacid CoA-transferase 1; CPT1, carnitine palmitoyltransferase 1; OA, oxaloacetate.

Recent discoveries in LEC metabolism have expanded current scientific knowledge on the lymphatic vasculature and lymphangiogenesis. Glycolysis, FAO, KBO, mitochondrial respiration, and LD autophagy are all utilized by LECs to support necessary processes underlying lymphatic development (Figure 2). Specifically, glycolysis is the main metabolic pathway utilized by LECs to generate ATP, driving lymphatic development. FAO has also been shown to support energy production when glycolysis is diminished, indicating the role of metabolic flexibility in maintaining energy homeostasis in LECs. Furthermore, FAO supports the generation of acetyl-CoA, which can be utilized as a substrate for epigenetic modification of *VEGFR3* in LECs via histone acetylation, thus driving lymphatic development. Additionally, KBO supports lymphatic development and is of particular interest as a potential therapeutic strategy to address lymphedema. Mitochondrial respiration plays a role in regulating LEC fate identity via epigenetic modification, and aids in the production of TCA cycle intermediates. Lastly, LD autophagy can be utilized to support FAO in LECs during lymphangiogenesis. Although it is established that LECs utilize the aforementioned metabolic pathways to support lymphatic development, several knowledge gaps remain to be addressed. For example, how are these metabolic processes regulated by different growth factors or cytokines during development and pathological conditions? How do different metabolic pathways interact and coordinate to enable LV growth? Under which physiological and/or pathological conditions do LECs shift their energy sources? Is there any metabolic crosstalk between LECs and their surrounding environment? Although much remains to be discovered mechanistically and clinically, enzymes involved in LEC metabolism are promising targets in the development of new treatments for human pathological conditions related to the lymphatic system.

## References

[1] Stacker SA Williams SP Karnezis T Shayan R Fox SB Achen MG. Lymphangiogenesis and lymphatic vessel remodelling in cancer. Nat Rev Cancer. (2014) 14(3):159–72. 10.1038/nrc3677 24561443

[2] Klotz L Norman S Vieira JM Masters M Rohling M Dube KN Cardiac lymphatics are heterogeneous in origin and respond to injury. Nature. (2015) 522(7554):62–7. 10.1038/nature14483 25992544 PMC4458138

[3] Vieira JM Norman S Villa Del Campo C Cahill TJ Barnette DN Gunadasa-Rohling M The cardiac lymphatic system stimulates resolution of inflammation following myocardial infarction. J Clin Invest. (2018) 128(8):3402–12. 10.1172/JCI97192 29985167 PMC6063482

[4] Chen L Hamrah P Cursiefen C Zhang Q Pytowski B Streilein JW Vascular endothelial growth factor receptor-3 mediates induction of corneal alloimmunity. Nat Med. (2004) 10(8):813–5. 10.1038/nm1078 15235599

[5] Brouillard P Witte MH Erickson RP Damstra RJ Becker C Quere I Primary lymphoedema. Nat Rev Dis Primers. (2021) 7(1):77. 10.1038/s41572-021-00309-7 34675250

[6] Dieterich LC Seidel CD Detmar M. Lymphatic vessels: new targets for the treatment of inflammatory diseases. Angiogenesis. (2014) 17(2):359–71. 10.1007/s10456-013-9406-1 24212981

[7] Pepper MS Skobe M. Lymphatic endothelium: morphological, molecular and functional properties. J Cell Biol. (2003) 163(2):209–13. 10.1083/jcb.200308082 14581448 PMC2173536

[8] Baluk P Fuxe J Hashizume H Romano T Lashnits E Butz S Functionally specialized junctions between endothelial cells of lymphatic vessels. J Exp Med. (2007) 204(10):2349–62. 10.1084/jem.20062596 17846148 PMC2118470

[9] Yao LC McDonald DM. Plasticity of airway lymphatics in development and disease. Adv Anat Embryol Cell Biol. (2014) 214:41–54. 10.1007/978-3-7091-1646-3_4 24276885 PMC3955364

[10] Null M Arbor TC Agarwal M. Anatomy, Lymphatic System. Treasure Island, FL: StatPearls (2024). 30020619

[11] Cruz de Casas P Knopper K Dey Sarkar R Kastenmuller W. Same yet different—how lymph node heterogeneity affects immune responses. Nat Rev Immunol. (2023). 10.1038/s41577-023-00965-8 38097778

[12] Escobedo N Oliver G. The lymphatic vasculature: its role in adipose metabolism and obesity. Cell Metab. (2017) 26(4):598–609. 10.1016/j.cmet.2017.07.020 28844882 PMC5629116

[13] Petrova TV Koh GY. Biological functions of lymphatic vessels. Science. (2020) 369(6500). 10.1126/science.aax4063 32646971

[14] Cao E Watt MJ Nowell CJ Quach T Simpson JS De Melo Ferreira V Mesenteric lymphatic dysfunction promotes insulin resistance and represents a potential treatment target in obesity. Nat Metab. (2021) 3(9):1175–88. 10.1038/s42255-021-00457-w 34545251

[15] Huang LH Elvington A Randolph GJ. The role of the lymphatic system in cholesterol transport. Front Pharmacol. (2015) 6:182. 10.3389/fphar.2015.00182 26388772 PMC4557107

[16] Jakus Z Gleghorn JP Enis DR Sen A Chia S Liu X Lymphatic function is required prenatally for lung inflation at birth. J Exp Med. (2014) 211(5):815–26. 10.1084/jem.20132308 24733830 PMC4010903

[17] Ahn JH Cho H Kim JH Kim SH Ham JS Park I Meningeal lymphatic vessels at the skull base drain cerebrospinal fluid. Nature. (2019) 572(7767):62–6. 10.1038/s41586-019-1419-5 31341278

[18] Louveau A Plog BA Antila S Alitalo K Nedergaard M Kipnis J. Understanding the functions and relationships of the glymphatic system and meningeal lymphatics. J Clin Invest. (2017) 127(9):3210–9. 10.1172/JCI90603 28862640 PMC5669566

[19] Makinen T Norrmen C Petrova TV. Molecular mechanisms of lymphatic vascular development. Cell Mol Life Sci. (2007) 64(15):1915–29. 10.1007/s00018-007-7040-z 17458498 PMC11136057

[20] Carmeliet P. Mechanisms of angiogenesis and arteriogenesis. Nat Med. (2000) 6(4):389–95. 10.1038/74651 10742145

[21] Trimm E Red-Horse K. Vascular endothelial cell development and diversity. Nat Rev Cardiol. (2023) 20(3):197–210. 10.1038/s41569-022-00770-1 36198871 PMC9533272

[22] Oliver G Srinivasan RS. Endothelial cell plasticity: how to become and remain a lymphatic endothelial cell. Development. (2010) 137(3):363–72. 10.1242/dev.035360 20081185 PMC2858906

[23] Wigle JT Oliver G. Prox1 function is required for the development of the murine lymphatic system. Cell. (1999) 98(6):769–78. 10.1016/S0092-8674(00)81511-1 10499794

[24] Hu Z Zhao X Wu Z Qu B Yuan M Xing Y Lymphatic vessel: origin, heterogeneity, biological functions, and therapeutic targets. Signal Transduct Target Ther. (2024) 9(1):9. 10.1038/s41392-023-01723-x 38172098 PMC10764842

[25] Joukov V Pajusola K Kaipainen A Chilov D Lahtinen I Kukk E A novel vascular endothelial growth factor, VEGF-C, is a ligand for the Flt4 (VEGFR-3) and KDR (VEGFR-2) receptor tyrosine kinases. EMBO J. (1996) 15(7):1751. 10.1002/j.1460-2075.1996.tb00521.x 8612600 PMC450088

[26] Yang Y Garcia-Verdugo JM Soriano-Navarro M Srinivasan RS Scallan JP Singh MK Lymphatic endothelial progenitors bud from the cardinal vein and intersomitic vessels in mammalian embryos. Blood. (2012) 120(11):2340–8. 10.1182/blood-2012-05-428607 22859612 PMC3447786

[27] Srinivasan RS Geng X Yang Y Wang Y Mukatira S Studer M The nuclear hormone receptor coup-TFII is required for the initiation and early maintenance of Prox1 expression in lymphatic endothelial cells. Genes Dev. (2010) 24(7):696–707. 10.1101/gad.1859310 20360386 PMC2849126

[28] Hong YK Harvey N Noh YH Schacht V Hirakawa S Detmar M Prox1 is a master control gene in the program specifying lymphatic endothelial cell fate. Dev Dyn. (2002) 225(3):351–7. 10.1002/dvdy.10163 12412020

[29] Srinivasan RS Escobedo N Yang Y Interiano A Dillard ME Finkelstein D The Prox1-Vegfr3 feedback loop maintains the identity and the number of lymphatic endothelial cell progenitors. Genes Dev. (2014) 28(19):2175–87. 10.1101/gad.216226.113 25274728 PMC4180978

[30] Yu P Wu G Lee HW Simons M. Endothelial metabolic control of lymphangiogenesis. Bioessays. (2018) 40(6):e1700245. 10.1002/bies.201700245 29750374 PMC6237195

[31] De Bock K Georgiadou M Schoors S Kuchnio A Wong BW Cantelmo AR Role of PFKFB3-driven glycolysis in vessel sprouting. Cell. (2013) 154(3):651–63. 10.1016/j.cell.2013.06.037 23911327

[32] Vander Heiden MG Cantley LC Thompson CB. Understanding the warburg effect: the metabolic requirements of cell proliferation. Science. (2009) 324(5930):1029–33. 10.1126/science.1160809 19460998 PMC2849637

[33] Wilson JE. Isozymes of mammalian hexokinase: structure, subcellular localization and metabolic function. J Exp Biol. (2003) 206(Pt 12):2049–57. 10.1242/jeb.00241 12756287

[34] Robey RB Hay N. Mitochondrial hexokinases, novel mediators of the antiapoptotic effects of growth factors and akt. Oncogene. (2006) 25(34):4683–96. 10.1038/sj.onc.1209595 16892082

[35] DeWaal D Nogueira V Terry AR Patra KC Jeon SM Guzman G Hexokinase-2 depletion inhibits glycolysis and induces oxidative phosphorylation in hepatocellular carcinoma and sensitizes to metformin. Nat Commun. (2018) 9(1):446. 10.1038/s41467-017-02733-4 29386513 PMC5792493

[36] Miyamoto S Murphy AN Brown JH. Akt mediates mitochondrial protection in cardiomyocytes through phosphorylation of mitochondrial hexokinase-II. Cell Death Differ. (2008) 15(3):521–9. 10.1038/sj.cdd.4402285 18064042

[37] Sun L Shukair S Naik TJ Moazed F Ardehali H. Glucose phosphorylation and mitochondrial binding are required for the protective effects of hexokinases I and II. Mol Cell Biol. (2008) 28(3):1007–17. 10.1128/MCB.00224-07 18039843 PMC2223386

[38] Yu P Wilhelm K Dubrac A Tung JK Alves TC Fang JS FGF-dependent metabolic control of vascular development. Nature. (2017) 545(7653):224–8. 10.1038/nature22322 28467822 PMC5427179

[39] Xu Y An X Guo X Habtetsion TG Wang Y Xu X Endothelial PFKFB3 plays a critical role in angiogenesis. Arterioscler Thromb Vasc Biol. (2014) 34(6):1231–9. 10.1161/ATVBAHA.113.303041 24700124 PMC4120754

[40] Jiang H Zou Y Zhao J Li X Yang S Zhou X Pyruvate kinase M2 mediates glycolysis in the lymphatic endothelial cells and promotes the progression of lymphatic malformations. Am J Pathol. (2021) 191(1):204–15. 10.1016/j.ajpath.2020.10.003 33130045

[41] Lunt SY Vander Heiden MG. Aerobic glycolysis: meeting the metabolic requirements of cell proliferation. Annu Rev Cell Dev Biol. (2011) 27:441–64. 10.1146/annurev-cellbio-092910-154237 21985671

[42] Meng YM Jiang X Zhao X Meng Q Wu S Chen Y Hexokinase 2-driven glycolysis in pericytes activates their contractility leading to tumor blood vessel abnormalities. Nat Commun. (2021) 12(1):6011. 10.1038/s41467-021-26259-y 34650057 PMC8517026

[43] Armulik A Genove G Betsholtz C. Pericytes: developmental, physiological, and pathological perspectives, problems, and promises. Dev Cell. (2011) 21(2):193–215. 10.1016/j.devcel.2011.07.001 21839917

[44] Holm A Heumann T Augustin HG. Microvascular mural cell organotypic heterogeneity and functional plasticity. Trends Cell Biol. (2018) 28(4):302–16. 10.1016/j.tcb.2017.12.002 29307447

[45] Gerhardt H Betsholtz C. Endothelial-pericyte interactions in angiogenesis. Cell Tissue Res. (2003) 314(1):15–23. 10.1007/s00441-003-0745-x 12883993

[46] Gaengel K Genove G Armulik A Betsholtz C. Endothelial-mural cell signaling in vascular development and angiogenesis. Arterioscler Thromb Vasc Biol. (2009) 29(5):630–8. 10.1161/ATVBAHA.107.161521 19164813

[47] Andrae J Gallini R Betsholtz C. Role of platelet-derived growth factors in physiology and medicine. Genes Dev. (2008) 22(10):1276–312. 10.1101/gad.1653708 18483217 PMC2732412

[48] Lindahl P Johansson BR Leveen P Betsholtz C. Pericyte loss and microaneurysm formation in PDGF-B-deficient mice. Science. (1997) 277(5323):242–5. 10.1126/science.277.5323.242 9211853

[49] Bergers G Song S. The role of pericytes in blood-vessel formation and maintenance. Neuro Oncol. (2005) 7(4):452–64. 10.1215/S1152851705000232 16212810 PMC1871727

[50] Jiang Z Zhou J Li L Liao S He J Zhou S Pericytes in the tumor microenvironment. Cancer Lett. (2023) 556:216074. 10.1016/j.canlet.2023.216074 36682706

[51] Joyce JA Pollard JW. Microenvironmental regulation of metastasis. Nat Rev Cancer. (2009) 9(4):239–52. 10.1038/nrc2618 19279573 PMC3251309

[52] Hanahan D Weinberg RA. Hallmarks of cancer: the next generation. Cell. (2011) 144(5):646–74. 10.1016/j.cell.2011.02.013 21376230

[53] Abramsson A Berlin O Papayan H Paulin D Shani M Betsholtz C. Analysis of mural cell recruitment to tumor vessels. Circulation. (2002) 105(1):112–7. 10.1161/hc0102.101437 11772885

[54] Nolan-Stevaux O Truitt MC Pahler JC Olson P Guinto C Lee DC Differential contribution to neuroendocrine tumorigenesis of parallel egfr signaling in cancer cells and pericytes. Genes Cancer. (2010) 1(2):125–41. 10.1177/1947601909358722 20975924 PMC2958675

[55] Song N Huang Y Shi H Yuan S Ding Y Song X Overexpression of platelet-derived growth factor-BB increases tumor pericyte content via stromal-derived factor-1alpha/CXCR4 axis. Cancer Res. (2009) 69(15):6057–64. 10.1158/0008-5472.CAN-08-2007 19584297

[56] Kern TS Tang J Mizutani M Kowluru RA Nagaraj RH Romeo G Response of capillary cell death to aminoguanidine predicts the development of retinopathy: comparison of diabetes and galactosemia. Invest Ophthalmol Vis Sci. (2000) 41(12):3972–8. 11053301

[57] Wilhelm K Happel K Eelen G Schoors S Oellerich MF Lim R FOXO1 Couples metabolic activity and growth state in the vascular endothelium. Nature. (2016) 529(7585):216–20. 10.1038/nature16498 26735015 PMC5380221

[58] Dong Y Tu R Liu H Qing G. Regulation of cancer cell metabolism: oncogenic MYC in the driver’s seat. Signal Transduct Target Ther. (2020) 5(1):124. 10.1038/s41392-020-00235-2 32651356 PMC7351732

[59] Lopaschuk GD Ussher JR Folmes CD Jaswal JS Stanley WC. Myocardial fatty acid metabolism in health and disease. Physiol Rev. (2010) 90(1):207–58. 10.1152/physrev.00015.2009 20086077

[60] Ma Y Temkin SM Hawkridge AM Guo C Wang W Wang XY Fatty acid oxidation: an emerging facet of metabolic transformation in cancer. Cancer Lett. (2018) 435:92–100. 10.1016/j.canlet.2018.08.006 30102953 PMC6240910

[61] Wong BW Wang X Zecchin A Thienpont B Cornelissen I Kalucka J The role of fatty acid beta-oxidation in lymphangiogenesis. Nature. (2017) 542(7639):49–54. 10.1038/nature21028 28024299

[62] Raud B Roy DG Divakaruni AS Tarasenko TN Franke R Ma EH Etomoxir actions on regulatory and memory T cells are independent of Cpt1a-mediated fatty acid oxidation. Cell Metab. (2018) 28(3):504–15.e7. 10.1016/j.cmet.2018.06.002 30043753 PMC6747686

[63] Yao CH Liu GY Wang R Moon SH Gross RW Patti GJ. Identifying off-target effects of etomoxir reveals that carnitine palmitoyltransferase I is essential for cancer cell proliferation independent of beta-oxidation. PLoS Biol. (2018) 16(3):e2003782. 10.1371/journal.pbio.2003782 29596410 PMC5892939

[64] Ko CW Qu J Black DD Tso P. Regulation of intestinal lipid metabolism: current concepts and relevance to disease. Nat Rev Gastroenterol Hepatol. (2020) 17(3):169–83. 10.1038/s41575-019-0250-7 32015520

[65] Cifarelli V Appak-Baskoy S Peche VS Kluzak A Shew T Narendran R Visceral obesity and insulin resistance associate with CD36 deletion in lymphatic endothelial cells. Nat Commun. (2021) 12(1):3350. 10.1038/s41467-021-23808-3 34099721 PMC8184948

[66] Zhang F Zarkada G Han J Li J Dubrac A Ola R Lacteal junction zippering protects against diet-induced obesity. Science. (2018) 361(6402):599–603. 10.1126/science.aap9331 30093598 PMC6317738

[67] Etchegaray JP Mostoslavsky R. Interplay between metabolism and epigenetics: a nuclear adaptation to environmental changes. Mol Cell. (2016) 62(5):695–711. 10.1016/j.molcel.2016.05.029 27259202 PMC4893201

[68] Verdin E Ott M. 50 years of protein acetylation: from gene regulation to epigenetics, metabolism and beyond. Nat Rev Mol Cell Biol. (2015) 16(4):258–64. 10.1038/nrm3931 25549891

[69] Lee KK Workman JL. Histone acetyltransferase complexes: one size doesn't fit all. Nat Rev Mol Cell Biol. (2007) 8(4):284–95. 10.1038/nrm2145 17380162

[70] Wellen KE Hatzivassiliou G Sachdeva UM Bui TV Cross JR Thompson CB. ATP-citrate lyase links cellular metabolism to histone acetylation. Science. (2009) 324(5930):1076–80. 10.1126/science.1164097 19461003 PMC2746744

[71] Tropberger P Pott S Keller C Kamieniarz-Gdula K Caron M Richter F Regulation of transcription through acetylation of H3K122 on the lateral surface of the histone octamer. Cell. (2013) 152(4):859–72. 10.1016/j.cell.2013.01.032 23415232

[72] Ichise T Yoshida N Ichise H. Ras/MAPK signaling modulates VEGFR-3 expression through ets-mediated p300 recruitment and histone acetylation on the Vegfr3 gene in lymphatic endothelial cells. PLoS One. (2012) 7(12):e51639. 10.1371/journal.pone.0051639 23284731 PMC3524184

[73] Schoors S Bruning U Missiaen R Queiroz KC Borgers G Elia I Fatty acid carbon is essential for dNTP synthesis in endothelial cells. Nature. (2015) 520(7546):192–7. 10.1038/nature14362 25830893 PMC4413024

[74] Song H Zhu J Li P Han F Fang L Yu P. Metabolic flexibility maintains proliferation and migration of FGFR signaling-deficient lymphatic endothelial cells. J Biol Chem. (2021) 297(4):101149. 10.1016/j.jbc.2021.101149 34473994 PMC8498002

[75] Montenegro-Navarro N Garcia-Baez C Garcia-Caballero M. Molecular and metabolic orchestration of the lymphatic vasculature in physiology and pathology. Nat Commun. (2023) 14(1):8389. 10.1038/s41467-023-44133-x 38104163 PMC10725466

[76] Tieu K Perier C Caspersen C Teismann P Wu DC Yan SD D-beta-hydroxybutyrate rescues mitochondrial respiration and mitigates features of Parkinson disease. J Clin Invest. (2003) 112(6):892–901. 10.1172/JCI200318797 12975474 PMC193668

[77] Zhang S Xie C. The role of OXCT1 in the pathogenesis of cancer as a rate-limiting enzyme of ketone body metabolism. Life Sci. (2017) 183:110–5. 10.1016/j.lfs.2017.07.003 28684065

[78] Garcia-Caballero M Zecchin A Souffreau J Truong ACK Teuwen LA Vermaelen W Role and therapeutic potential of dietary ketone bodies in lymph vessel growth. Nat Metab. (2019) 1(7):666–75. 10.1038/s42255-019-0087-y 32694649

[79] Klimova T Chandel NS. Mitochondrial complex III regulates hypoxic activation of HIF. Cell Death Differ. (2008) 15(4):660–6. 10.1038/sj.cdd.4402307 18219320

[80] Barel O Shorer Z Flusser H Ofir R Narkis G Finer G Mitochondrial complex III deficiency associated with a homozygous mutation in UQCRQ. Am J Hum Genet. (2008) 82(5):1211–6. 10.1016/j.ajhg.2008.03.020 18439546 PMC2427202

[81] Ma W Gil HJ Liu X Diebold LP Morgan MA Oxendine-Burns MJ Mitochondrial respiration controls the Prox1-Vegfr3 feedback loop during lymphatic endothelial cell fate specification and maintenance. Sci Adv. (2021) 7(18). 10.1126/sciadv.abe7359 PMC8087398 33931446

[82] Schaaf MB Houbaert D Mece O Agostinis P. Autophagy in endothelial cells and tumor angiogenesis. Cell Death Differ. (2019) 26(4):665–79. 10.1038/s41418-019-0287-8 30692642 PMC6460396

[83] Nussenzweig SC Verma S Finkel T. The role of autophagy in vascular biology. Circ Res. (2015) 116(3):480–8. 10.1161/CIRCRESAHA.116.303805 25634971 PMC4313568

[84] Jarc E Petan T. Lipid droplets and the management of cellular stress. Yale J Biol Med. (2019) 92(3):435–52. 31543707 PMC6747940

[85] Mece O Houbaert D Sassano ML Durre T Maes H Schaaf M Lipid droplet degradation by autophagy connects mitochondria metabolism to Prox1-driven expression of lymphatic genes and lymphangiogenesis. Nat Commun. (2022) 13(1):2760. 10.1038/s41467-022-30490-6 35589749 PMC9120506

[86] Olzmann JA Carvalho P. Dynamics and functions of lipid droplets. Nat Rev Mol Cell Biol. (2019) 20(3):137–55. 10.1038/s41580-018-0085-z 30523332 PMC6746329

[87] Zechner R Madeo F Kratky D. Cytosolic lipolysis and lipophagy: two sides of the same coin. Nat Rev Mol Cell Biol. (2017) 18(11):671–84. 10.1038/nrm.2017.76 28852221

[88] Sleigh BC Manna B. Lymphedema. Treasure Island, FL: StatPearls (2024). 30725924

[89] Slater HC Gambhir M Parham PE Michael E. Modelling co-infection with malaria and lymphatic filariasis. PLoS Comput Biol. (2013) 9(6):e1003096. 10.1371/journal.pcbi.1003096 23785271 PMC3681634

[90] DiSipio T Rye S Newman B Hayes S. Incidence of unilateral arm lymphoedema after breast cancer: a systematic review and meta-analysis. Lancet Oncol. (2013) 14(6):500–15. 10.1016/S1470-2045(13)70076-7 23540561

[91] Hayes SC Janda M Ward LC Reul-Hirche H Steele ML Carter J Lymphedema following gynecological cancer: results from a prospective, longitudinal cohort study on prevalence, incidence and risk factors. Gynecol Oncol. (2017) 146(3):623–9. 10.1016/j.ygyno.2017.06.004 28624154

[92] Warren AG Brorson H Borud LJ Slavin SA. Lymphedema: a comprehensive review. Ann Plast Surg. (2007) 59(4):464–72. 10.1097/01.sap.0000257149.42922.7e 17901744

[93] Granzow JW. Lymphedema surgery: the current state of the art. Clin Exp Metastasis. (2018) 35(5-6):553–8. 10.1007/s10585-018-9897-7 29980891

[94] Schneider M Ny A Ruiz de Almodovar C Carmeliet P. A new mouse model to study acquired lymphedema. PLoS Med. (2006) 3(7):e264. 10.1371/journal.pmed.0030264 16842020 PMC1513046

[95] Tian W Rockson SG Jiang X Kim J Begaye A Shuffle EM Leukotriene B(4) antagonism ameliorates experimental lymphedema. Sci Transl Med. (2017) 9(389). 10.1126/scitranslmed.aal3920 28490670

[96] Zampell JC Yan A Elhadad S Avraham T Weitman E Mehrara BJ. CD4(+) cells regulate fibrosis and lymphangiogenesis in response to lymphatic fluid stasis. PLoS One. (2012) 7(11):e49940. 10.1371/journal.pone.0049940 23185491 PMC3502174

[97] Farag TI Teebi AS. Autosomal recessive inheritance of a syndrome of hypertelorism, hypospadias, and tetralogy of fallot? Am J Med Genet. (1990) 35(4):516–8. 10.1002/ajmg.1320350414 2333881

[98] Meek KM Knupp C. Corneal structure and transparency. Prog Retin Eye Res. (2015) 49:1–16. 10.1016/j.preteyeres.2015.07.001 26145225 PMC4655862

[99] Azar DT. Corneal angiogenic privilege: angiogenic and antiangiogenic factors in corneal avascularity, vasculogenesis, and wound healing (an American ophthalmological society thesis). Trans Am Ophthalmol Soc. (2006) 104:264–302. 17471348 PMC1809914

[100] Patnam M Dommaraju SR Masood F Herbst P Chang JH Hu WY Lymphangiogenesis guidance mechanisms and therapeutic implications in pathological states of the cornea. Cells. (2023) 12(2):319. 10.3390/cells12020319 36672254 PMC9856498

[101] Yang JF Walia A Huang YH Han KY Rosenblatt MI Azar DT Understanding lymphangiogenesis in knockout models, the cornea, and ocular diseases for the development of therapeutic interventions. Surv Ophthalmol. (2016) 61(3):272–96. 10.1016/j.survophthal.2015.12.004 26706194 PMC4835265

[102] Cursiefen C Cao J Chen L Liu Y Maruyama K Jackson D Inhibition of hemangiogenesis and lymphangiogenesis after normal-risk corneal transplantation by neutralizing VEGF promotes graft survival. Invest Ophthalmol Vis Sci. (2004) 45(8):2666–73. 10.1167/iovs.03-1380 15277490

[103] Bachmann BO Bock F Wiegand SJ Maruyama K Dana MR Kruse FE Promotion of graft survival by vascular endothelial growth factor a neutralization after high-risk corneal transplantation. Arch Ophthalmol. (2008) 126(1):71–7. 10.1001/archopht.126.1.71 18195221

[104] Bachmann BO Luetjen-Drecoll E Bock F Wiegand SJ Hos D Dana R Transient postoperative vascular endothelial growth factor (VEGF)-neutralisation improves graft survival in corneas with partly regressed inflammatory neovascularisation. Br J Ophthalmol. (2009) 93(8):1075–80. 10.1136/bjo.2008.145128 19224901

[105] Dietrich T Bock F Yuen D Hos D Bachmann BO Zahn G Cutting edge: lymphatic vessels, not blood vessels, primarily mediate immune rejections after transplantation. J Immunol. (2010) 184(2):535–9. 10.4049/jimmunol.0903180 20018627 PMC4725297

[106] Yin N Zhang N Xu J Shi Q Ding Y Bromberg JS. Targeting lymphangiogenesis after islet transplantation prolongs islet allograft survival. Transplantation. (2011) 92(1):25–30. 10.1097/TP.0b013e31821d2661 21508896 PMC3703312

[107] Yan H Yuan J Peng R Wang T Deng J Li W The blockade of vascular endothelial growth factor C effectively inhibits corneal lymphangiogenesis and promotes allograft survival. J Ocul Pharmacol Ther. (2015) 31(9):546–54. 10.1089/jop.2015.0007 26172526

[108] Gurnani B Czyz CN Mahabadi N Havens SJ. Corneal Graft Rejection. Treasure Island, FL: StatPearls (2024). 30085585

[109] Detry B Blacher S Erpicum C Paupert J Maertens L Maillard C Sunitinib inhibits inflammatory corneal lymphangiogenesis. Invest Ophthalmol Vis Sci. (2013) 54(5):3082–93. 10.1167/iovs.12-10856 23580490

[110] Chen WS Cao Z Sugaya S Lopez MJ Sendra VG Laver N Pathological lymphangiogenesis is modulated by galectin-8-dependent crosstalk between podoplanin and integrin-associated VEGFR-3. Nat Commun. (2016) 7:11302. 10.1038/ncomms11302 27066737 PMC4832077

